# Transorbital Approach to Surgical Resection of a Far-Lateral Frontal Sinus Osteoma

**DOI:** 10.1055/a-2831-9153

**Published:** 2026-03-20

**Authors:** Leah T. Rosen, Gabriella Schmuter, Sruti Akula, Shanlee Stevens, Celestine Gregerson, Michael J. Ye, Abtin Tabaee, Kyle J. Godfrey

**Affiliations:** 1 Israel Englander Department of Ophthalmology, NewYork-Presbyterian Weill Cornell Medicine, New York, New York, United States; 2 Department of Otolaryngology, NewYork-Presbyterian Weill Cornell Medicine, New York, New York, United States; 3 Department of Neurological Surgery, NewYork-Presbyterian Weill Cornell Medicine, New York, New York, United States; 4 University of California, Los Angeles, Stein Eye Institue

**Keywords:** TOA, LTOA, minimally invasive, transorbital approach

## Abstract

A 21-year-old male presented with chronic left-sided rhinosinusitis and was found to have a left frontal sinus mass on maxillofacial computed tomography (CT). The mass measured approximately 1.9 × 1.3 × 2.3 cm in size and was contiguous with the intrasinus septum and anterior table of the frontal sinus. Imaging features were consistent with osteoma. An endoscopic endonasal approach resulted in subtotal resection of the mass. Eleven months later, the patient underwent a combined left transorbital and endoscopic surgical resection with oculofacial plastic surgery and otolaryngology. The mass was resected in its entirety, and histopathology confirmed an osteoma. This is one of the few reports of a unilateral transorbital frontal sinus osteoma resection and highlights the utility of the transorbital approach for minimally invasive access to the far-lateral frontal sinus.

Benign neoplasms of the paranasal sinuses and skull base pose a unique challenge for surgeons. These lesions are often asymptomatic; however, local expansion and associated mass effect may cause symptoms warranting surgical removal. Symptoms may include restricted extraocular movements or vision loss when the neoplasm involves the skull base, and interdisciplinary collaboration with otolaryngology, neurology, and/or neurosurgery may be essential for optimal care coordination and clinical outcomes. 1

Evaluation of these neoplasms includes thin-sectioned multiplanar computed tomography (CT) or magnetic resonance imaging (MRI) and potentially endoscopic visualization if the mass is within the sinuses. Common indications for surgical resection of sinonasal osteoma include paranasal sinus outflow tract obstruction, extrasinus expansion of the lesion (e.g., orbit, intracranial), evidence of enlargement on serial imaging, and clinical or radiographic concern for malignant pathology (e.g., osteosarcoma). 2 The size, radiographic features, location, and mass effect of the lesion are considered when determining the surgical approach. For select neoplasms in the paranasal sinuses, endoscopic surgery alone is effective. However, the far-lateral frontal sinus can be challenging to access, and an endoscopic endonasal approach may not allow for total resection. 3 Additional factors, including tissue morphology (e.g., bony lesions) and invasiveness of the surrounding neurovascular structures, may limit the completeness of resection endonasally.

We present the case of a far-lateral frontal sinus neoplasm for which initial endonasal endoscopic surgery achieved a subtotal resection. A lateral transorbital surgical approach was then utilized, in collaboration with oculofacial plastic surgery and otolaryngology, which resulted in complete resection of the mass with no intermediate-term morbidity, demonstrating the utility of this interdisciplinary approach. This report adhered to the tenets of the Declaration of Helsinki and is in accordance with the Health Insurance Portability and Accountability Act (HIPAA). Patient consent has been obtained for this case report and the publication of its included images.

## Case Presentation

**Fig. 1:**
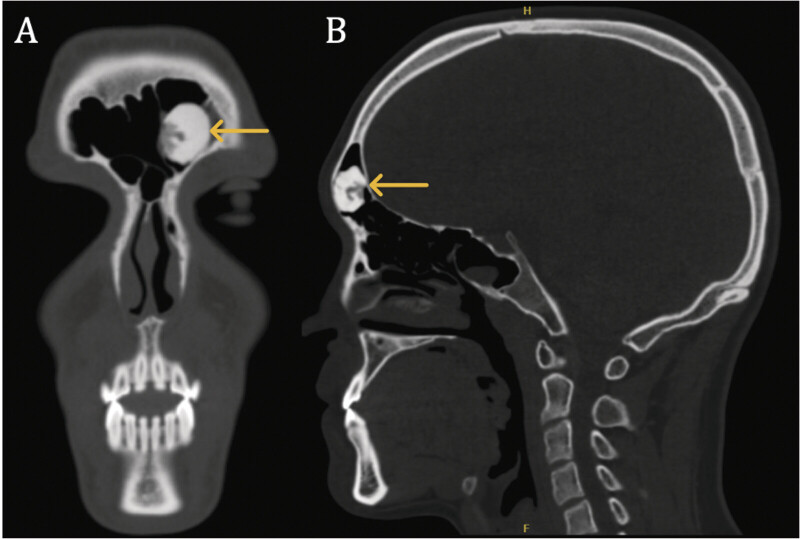
Preoperative coronal ( **A** ) and sagittal ( **B** ) CT maxillofacial imaging demonstrating an irregularly ovoid mass contiguous with the intrasinus septum and anterior table of the frontal sinus (identified via yellow arrows). CT, computed tomography.

A 21-year-old male presented to the otolaryngology clinic for chronic, intermittent left-sided nasal congestion. Maxillofacial CT revealed a bony mass in the left frontal sinus with irregularly ovoid morphology, measuring 1.9 × 1.3 × 1.2 cm, that was contiguous with both the intrasinus septum and anterior table of the frontal sinus ( Fig. 1 ). The overall radiographic appearance of the mass was consistent with an osteoma.

The indications for surgical resection in this patient included the large size of the osteoma with concern for frontal recess outflow tract obstruction, radiographic evidence of thinning of the posterior frontal table with concern for future intracranial spread. Further, the presence of areas of “immature” osteoma on imaging in our relatively young patient raised the concern that surgery would become inevitable at some point over his lifetime. The patient initially underwent a combined nasal septoplasty and endoscopic sinus surgery with otolaryngology. A modified endoscopic, transnasal Lothrop procedure, or Draf III, was performed. However, due to the far lateral position and bony nature of the lesion, the superior resection was incomplete.

**Fig. 2:**
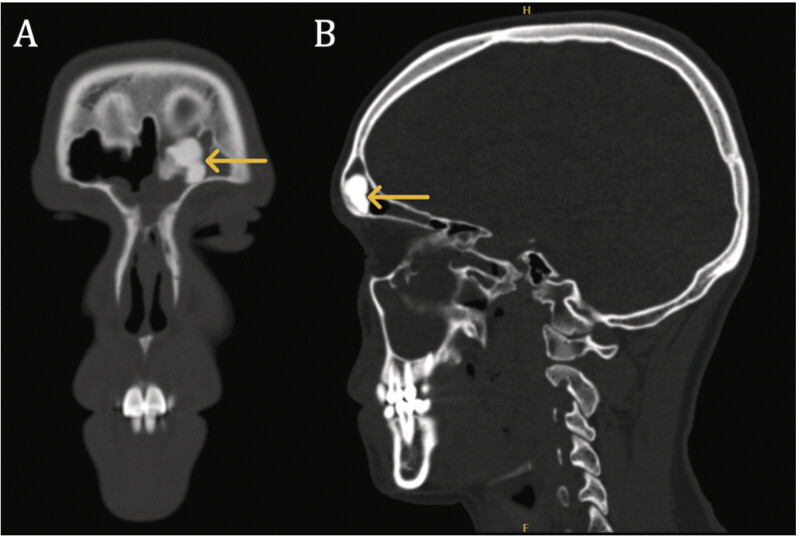
Coronal ( **A** ) and sagittal ( **B** ) CT maxillofacial images demonstrating the residual frontal sinus mass (yellow arrows) after initial subtotal endonasal endoscopic resection. CT, computed tomography.

**Fig. 3:**
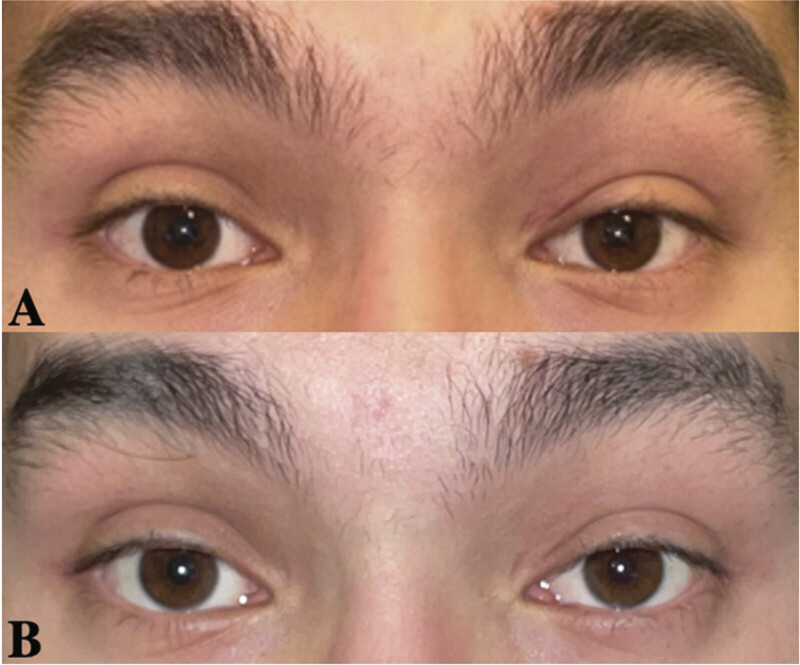
External photographs of the patient, preoperative ( **A** ) and at 6-month postoperative visit ( **B** ).

Follow-up CT performed 4 months later confirmed residual left frontal sinus mass ( Fig. 2 ). At this point, the patient was counseled on other surgical approaches, including consideration of a frontal sinus osteoplastic flap versus a lateral transorbital approach (LTOA). Given the patient's potential for receding hairline and the potential for aesthetically unacceptable scarring, a coronal-based approach was considered suboptimal, and the decision was made to pursue an LTOA. On examination, visual function, extraocular motility, and facial sensation were all within normal limits. There was no proptosis or facial asymmetry ( Fig. 3 ).

**Fig. 4:**
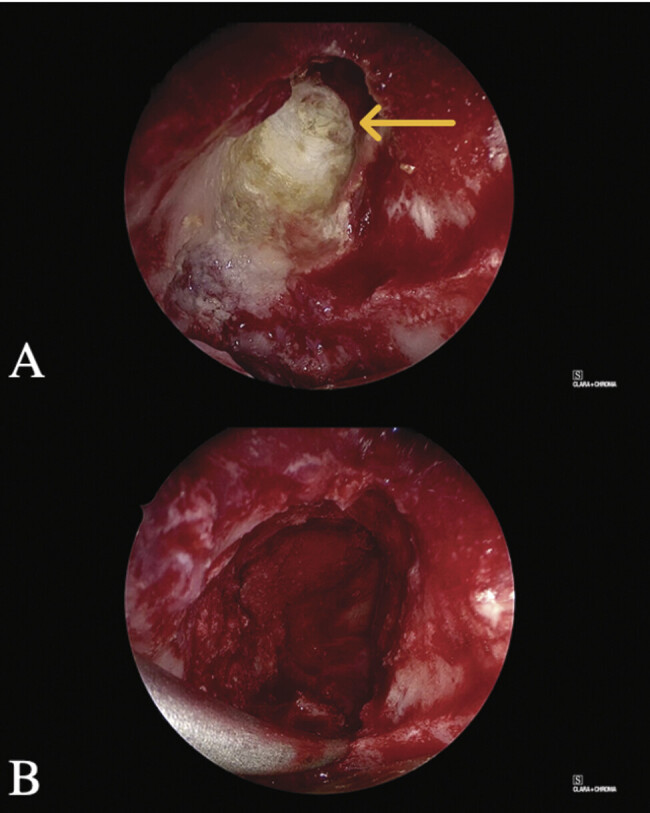
Intraoperative endoscopic photograph of the frontal sinus mass (yellow arrow) before ( **A** ) and after ( **B** ) resection.

An eyelid crease-based LTOA was performed on the left side. A lid crease incision was made, the orbicularis oculi muscle was divided, and a preseptal plane was then carried to the orbital rim. The arcus marginalis was incised while taking care to preserve the superomedial neurovascular bundles. A periosteal elevator was used to develop a subperiosteal plane, which was then carried posteriorly into the orbit to identify the superior orbital fissure. The position and margins of the mass were confirmed using stereotactic navigation, and the orbital roof was opened surrounding the base of the osteoma using a diamond burr and Kerrison Rongeurs. An angled endoscope provided direct visualization of the osteoma, posterior frontal sinus, and skull base. An angled 4-mm diamond burr was used to drill out the osteoma. The posterior table was visualized, and care was taken to avoid entering the anterior cranial fossa. With the assistance of combined endoscopic transorbital and endoscopic endonasal visualization, a resection plane was delineated, and the bony mass was excised in its entirety ( Fig. 4 ). Endoscopic visualization via transorbital and endonasal approaches confirmed complete gross tumor resection. Histopathological examination of the specimen confirmed osteoma.

**Fig. 5:**
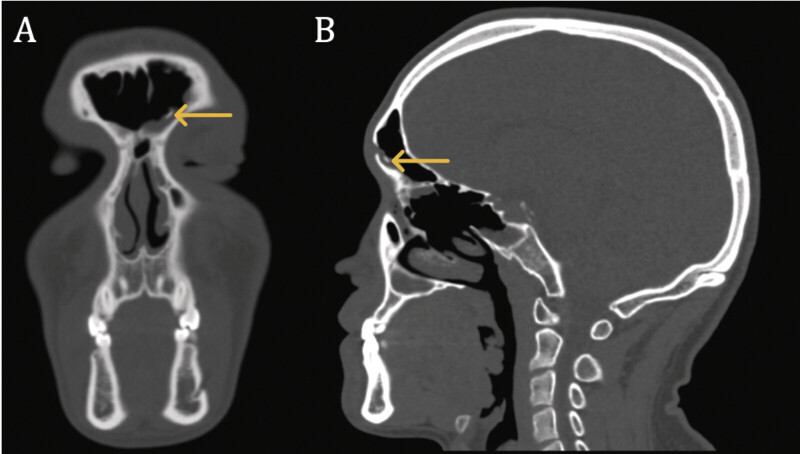
Coronal ( **A** ) and sagittal ( **B** ) CT maxillofacial images at postoperative month 6, demonstrating no definitive residual osteoma. There is a small focus of calcification (yellow arrow) in the left frontal sinus, potentially representing postsurgical change. CT, computed tomography.

In the immediate postoperative period, extraocular motility was full, and visual function was at baseline. At 1-week follow-up, the patient noted moderate pain and diminished sensation in the V1 distribution on the left side of his face; however, visual acuity, pupils, and extraocular movements were at baseline. The V1 hypoesthesia resolved at subsequent follow-up after the surgery. At 6-month follow-up, a postoperative maxillofacial CT demonstrated normal postsurgical changes and no residual osteoma ( Fig. 5 ).

## Discussion

About 70% to 80% of paranasal sinus osteomas are found in the frontal sinus, as seen in our patient. 4 These masses are known to be benign, slow-growing, and usually asymptomatic. In most cases, the masses can be observed through periodic follow-up and surveillance imaging. If a paranasal sinus osteoma compresses surrounding structures or obstructs sinus drainage, it can result in symptoms such as headache, recurrent sinusitis, or proptosis. 3 In these cases, surgical resection is recommended. 1

Historically, frontal sinus osteoma resections have followed one of three surgical approaches: (1) An external approach (i.e., osteoplastic flap, Lynch frontoethmoidectomy), (2) an endoscopic approach (i.e., endoscopic frontal sinusotomy), or (3) a combination of both. 5 A recent systematic review found that the most common approach presently is endoscopic surgery alone (44.9% of cases), followed by the osteoplastic flap (36.9%). 3 The endoscopic approach has gained popularity over recent years as a less invasive surgical option associated with minimal damage to surrounding structures, less morbidity, and better visualization of the relevant paranasal sinus anatomy. 4 In scenarios such as the one presented in this report, when the endonasal endoscopic surgery results in a subtotal resection, a subsequent, traditional osteoplastic flap is recommended to provide a wide exposure and complete resection. However, external approaches are associated with more severe postoperative pain and numbness, higher morbidity, and inferior cosmetic outcomes when compared to endoscopic surgeries. 5 6

When considering an osteoplastic flap, the patient found the risk of potential scarring along the hairline from a bicoronal approach to be cosmetically undesirable. Moreover, the patient expressed a preference for the transorbital approach (TOA) due to its advantages of faster postoperative recovery, lower morbidity, and reduced postoperative pain compared to external skull-based procedures. 7 Overall, combining the endoscopic and transorbital approach afforded optimal ability to manage the extensive frontal, periorbital, and posterior frontal table aspects of the lesion, along with favorable cosmesis.

Challenges during the surgery included visualization and drilling angle, which were aided by the use of an angled endoscope, concurrent illumination and visualization from the endoscopic endonasal approach, and a 70-degree diamond drill. The angle of approach also required prolonged soft tissue retraction, and the patient's early postoperative hypoesthesia in the V1 distribution was suspected to be related to retraction in the region of the supraorbital neurovascular bundle, producing neuropraxia. This risk is minimized by minimizing retraction force and continuous duration. Sensation in this distribution normalized around 3 months postoperatively, providing further evidence for this hypothesis. Reconstruction of the orbital roof defect was considered but deferred in this case without evidence of postoperative morbidity.

To the best of the authors' knowledge, this is one of the first reports describing a unilateral transorbital resection of a far-lateral frontal sinus osteoma. A recent paper by Yasuda et al describes two cases of the frontal sinus osteoid osteoma and includes the surgical technique of one: A combined right endonasal/transorbital approach for a large frontal sinus lesion causing lateral displacement of the globe. 8 This case differs from ours in (a) clinical presentation of each patient (age, symptoms, exam findings), (b) specific location of the lesion, and (c) duration of radiological follow-up. Also, there are intraoperative distinctions, in that the case described by Yasuda et al involved the removal of bony parts of the frontal sinus and inferior wall of the orbit—which were preserved in our case presentation. Therefore, the cumulative literature on this topic remains limited and would benefit from the addition of other case descriptions from other institutions.

Our case demonstrates an additional role for the LTOA in the management of craniofacial surgical challenges, particularly minimally invasive access to the far lateral frontal sinus. Safe adoption will depend on appropriate case selection, interdisciplinary collaboration, and ongoing advancements in endoscopic surgical expertise. 9 10
